# Social cognitive and affective neuroscience: the college years

**DOI:** 10.1093/scan/nsad080

**Published:** 2024-01-04

**Authors:** Matthew D Lieberman

**Affiliations:** University of California, 5544 Pritzker Hall, Los Angeles 90095-1563, USA

When it comes to *Social Cognitive and Affective* Neuroscience (SCAN), I feel a lot like a parent. Like most parents, I have been with SCAN since its conception. In 2004, a group of us met with Paul Kidd from Oxford University Press during a pre-conference to discuss the idea of a journal devoted to social neuroscience. Most of the room was opposed to the idea because they thought it would isolate social neuroscience from social psychology and from cognitive neuroscience. My take was that we already were isolated, with cognitive neuroscience journals often uninterested in our questions and social psychology journals dubious of our methods. Paul later approached me about becoming the founding Editor-in-Chief (EIC) of a new journal after multiple other more appropriate senior people said no. I was only a fourth-year Assistant Professor who had no business being an EIC but I truly believed social neuroscience needed its own home where the reviewers would care about our questions *and* methods. So, I said yes. I suggested we broaden the journal to include affective neuroscience as well. This was both because affective neuroscience did not have a journal of its own and because I was terrified that we would not get enough submissions. I suggested the journal title of *Social Cognitive and Affective Neuroscience* because it had a catchy neuroscience acronym and Paul Kidd agreed. From there, I spent 2005 choosing fonts, article formats and requirements, and convincing Paul Thompson to let us use an image from his lab that still graces the cover of SCAN. I lured a very esteemed initial board of Associate Editors with a promise that I would do most of the work. I kept that promise and was the action editor on every manuscript for the first three years of SCAN’s existence. We were digital only from the beginning except that we published a physical copy of the first year of the journal in a single thick volume to use as promotional materials. I probably have the only copies of that left. We started out with four issues per year and could barely fill those pages for the first year. Labs only had one or two studies and they didn’t want to send them to a new journal that wasn’t indexed yet for impact factors. Thanks to the amazing growth in social and affective neuroscience as a field, SCAN grew as well, becoming a flagship journal for this amazing area of research. In my time as editor, SCAN has published more than 2200 articles, with more than 300 of these being cited more than 100 times. It has been an absolute honor to oversee the publication of so many extraordinary papers.

As with any parent, there comes a time to let a child leave the nest and spread its wings without you. Given that SCAN is now in its 19th year of publication (and 20th year of existing), it has reached the college years which seems like the right moment to step aside. As a result, this will be my last year as EIC of SCAN and I will step down in January 2025. Several years ago, I realized that many on the editorial board had been there since 2005 and it was time to bring on lots of new younger faculty to make this journal feel like it belongs to the next generation of social and affective neuroscientists. My stepping down is the final step in that process. It is ironic that when I started, I was too junior to be the EIC at SCAN and now I am stepping down because I am too senior. I hope many of you will apply to be the new EIC. It’s an extraordinary opportunity to help shape and nurture a field that we all love. I’m happy to speak to anyone who might be interested in the position. I look forward to having someone who has a new vision of SCAN for the next generation of our field.

But in the immortal words of Monty Python, ‘I’m not quite dead yet’. I do have a few things in motion before I leave this position. First, Shannon Burns will be editing a special issue on ‘SAN and functional near-infrared spectroscopy (fNIRS)’. I am strongly of the opinion that fNIRS needs to be a central player in our field going forward. So much of what we want to study cannot be studied while a person is lying alone in an MRI scanner. fNIRS allows us to study dyads and teams while they interact. The equipment fits in carry-on luggage and can run off of a battery, so even people completely off the grid can be scanned. fNIRS has its downsides for sure, but if you are interested in regions of the default mode network and care about more ecologically valid neuroimaging, fNIRS has some enormous upsides. In my lab, we have been developing techniques that go beyond neural synchrony to allow multiple brains to be analyzed as a single multi-hub entity. I hope that SCAN will be welcoming many more fNIRS papers in the future that do not merely try to replicate what can be done in an MRI scanner, but truly take advantage of the ability of fNIRS to make social neuroscience more social.

Next, for the first time ever, SCAN will begin considering manuscripts that do not have an explicit neuroscience component. This new article type is called ‘Original research: non-neuroscience’. Historically, we have only considered studies looking at the structure or function of the central nervous system as they relate to social or affective processes. We will now accept a particular variety of non-neuroscience papers—those that would be of particular interest to the SCAN audience. These papers will typically focus on tasks specifically used in social and affective neuroscience studies or speak to issues that are ‘inside baseball’ within social and affective neuroscience. For instance, the monetary incentive delay (MID) task was created to study reward processes in the brain and has almost always been used in this way. If a lab conducted behavioral studies to tease apart processes involved in the MID, this would be appropriate for this new article type at SCAN because our audience would likely be highly interested in these results. Similarly, if someone created a computational model of empathic accuracy that did not directly look at the brain but drew from fMRI studies in this area, that might still be appropriate as this is a topic of significant interest to SCAN readers. Or consider the debate over contributions of theory of mind and the mirror system to everyday success at making sense of people’s mental states. If a study was conducted with behavioral measures of mirror and mentalizing processes and used these indicators to predict success in social interactions, this too would likely fit the new ‘non-neuroscience article’ article type. We do not foresee this new article type being particularly common, but we have noticed over the years that social and affective neuroscience labs will occasionally submit this kind of manuscript to SCAN and historically we have desk rejected them even though they would be of interest. Now there is a formal mechanism for them to be considered. Our expectation is that successful papers in this category would likely still be talking about the brain and thus be different from papers submitted to non-neuroscience journals.

Finally, after nearly two decades as EIC, the last thing I want to do before I exit is help our field create institutional structures so that we can support and grow social and affective neuroscience over the next two decades. At University of California, Los Angeles, the social and affective neuroscience faculty have been working towards creating a formal Social and Affective Neuroscience (SAN) area of the psychology department. There are lots of people who needed to be convinced this is a good idea. Along the way, we have accumulated a lot of information that I think would be very relevant to SAN faculty at other departments working to grow and solidify the SAN footprint so that there continue to be new faculty slots for our trainees to be hired into. To this end, my last contribution as EIC will be a ‘Tools of the Trade’ piece at SCAN that will provide a blueprint for ways to grow our institutional presence going forward.

Those are my goals for my final year at SCAN. After that, I look forward to cheering on the next editorial team at SCAN that will take us into the future.

